# CARD9 Signaling, Inflammation, and Diseases

**DOI:** 10.3389/fimmu.2022.880879

**Published:** 2022-03-30

**Authors:** Xuanyou Liu, Bimei Jiang, Hong Hao, Zhenguo Liu

**Affiliations:** ^1^ Center for Precision Medicine and Division of Cardiovascular Medicine, Department of Medicine, School of Medicine, University of Missouri, Columbia, MO, United States; ^2^ Department of Medical Pharmacology and Physiology, School of Medicine, University of Missouri, Columbia, MO, United States; ^3^ Department of Pathophysiology, Central South University, Changsha, China

**Keywords:** CARD9, cytokines, oxidative stress, autophagy, CARD9-associated diseases, tumor, cardiovascular diseases

## Abstract

Caspase-recruitment domain 9 (CARD9) protein is expressed in many cells especially in immune cells, and is critically involved in the function of the innate and adaptive immune systems through extensive interactions between CARD9 and other signaling molecules including NF-κB and MAPK. CARD9-mediated signaling plays a central role in regulating inflammatory responses and oxidative stress through the productions of important cytokines and chemokines. Abnormalities of CARD9 and CARD9 signaling or CARD9 mutations or polymorphism are associated with a variety of pathological conditions including infections, inflammation, and autoimmune disorders. This review focuses on the function of CARD9 and CARD9-mediated signaling pathways, as well as interactions with other important signaling molecules in different cell types and the relations to specific disease conditions including inflammatory diseases, infections, tumorigenesis, and cardiovascular pathologies.

## Introduction

Pattern recognition receptors (PRRs) in immune cells play a critical role in immune responses to infections of fungi, bacteria and other pathogens. There are four major types of PRRs: toll-like receptors (TLRs), c-type lectin receptors (CLRs), retinoic acid-inducible gene (RIG)-I-like receptors (RLRs), and NOD-like receptors (NLRs) (1, 2). TLRs and CLRs are transmembrane proteins for extracellular signaling, while RLRs and NLRs are cytoplasmic proteins for intracellular signaling (2). Following ligation of PRRs, a chain of intracellular signaling modules start to transmit the upstream signals to the downstream effector molecules. Caspase-recruitment domain 9 (CARD9) protein, one of the key intracellular modules, is mainly expressed in myeloid cells, especially in macrophages and dendritic cells (DCs), but also in cardiomyocytes and endothelial cells (3, 4). CARD9 expression can be found in almost all organ systems including spleen, lung, heart, brain, peripheral blood, and bone marrow (5–7). CARD9 is critically involved in both innate and adaptive immune responses to infections with fungi, bacteria, and viruses, and closely associated with infiltration and activation of immune cells and productions of pro-inflammatory cytokines (8, 9). Inflammation leads to macrophage invasion, activation, and release of cytokines, triggering oxidative stress and subsequent tissue dysfunction through a positive feedback loop that propagates oxidative stress and inflammation (10, 11).

Reactive oxygen species (ROS) formation and oxidative stress are closely related to the development and progression of a variety of diseases including infection, inflammatory diseases, tumors, and cardiovascular diseases (CVDs) (12–14). The critical roles of CARD9 in cytokines/chemokines secretion and ROS production in different cells and organ systems naturally connect CARD9 with the disease conditions associated with ROS and inflammation (4, 9, 15, 16). Mutation and genetic polymorphisms of CARD9 are reported to be correlated with a wide range of infectious diseases in human (17–19). In addition, CARD9 has been shown to be closely associated with CVDs, tumor formation and metastasis (20, 21). Thus, CARD9 could be a potential target for prevention and treatment of a variety of diseases including infections, CVDs, and cancer.

However, CARD9 signaling is very complex, and there are apparent inconsistencies in the roles of CARD9 signaling in the same pathological conditions including cardiac ischemia/reperfusion (I/R) injury and atherosclerosis for largely undefined mechanism(s). Thus, it is important to understand the complex and diverse roles of CARD9 signaling in the development and progression of specific disease conditions and related mechanisms to optimize the management with optimal outcomes of these conditions. In this review, we discuss the current knowledge on the diverse roles and molecular mechanisms of CARD9 signaling in the function of different cell types, the productions of cytokines/chemokines, adaptive immunity, oxidative stress, autophagy, and the development of a variety of diseases, hoping to provide a comprehensive understanding of the complex nature of CARD9 signaling in cell function and disease development.

## Biochemistry of CARD9

**Figure 1:**
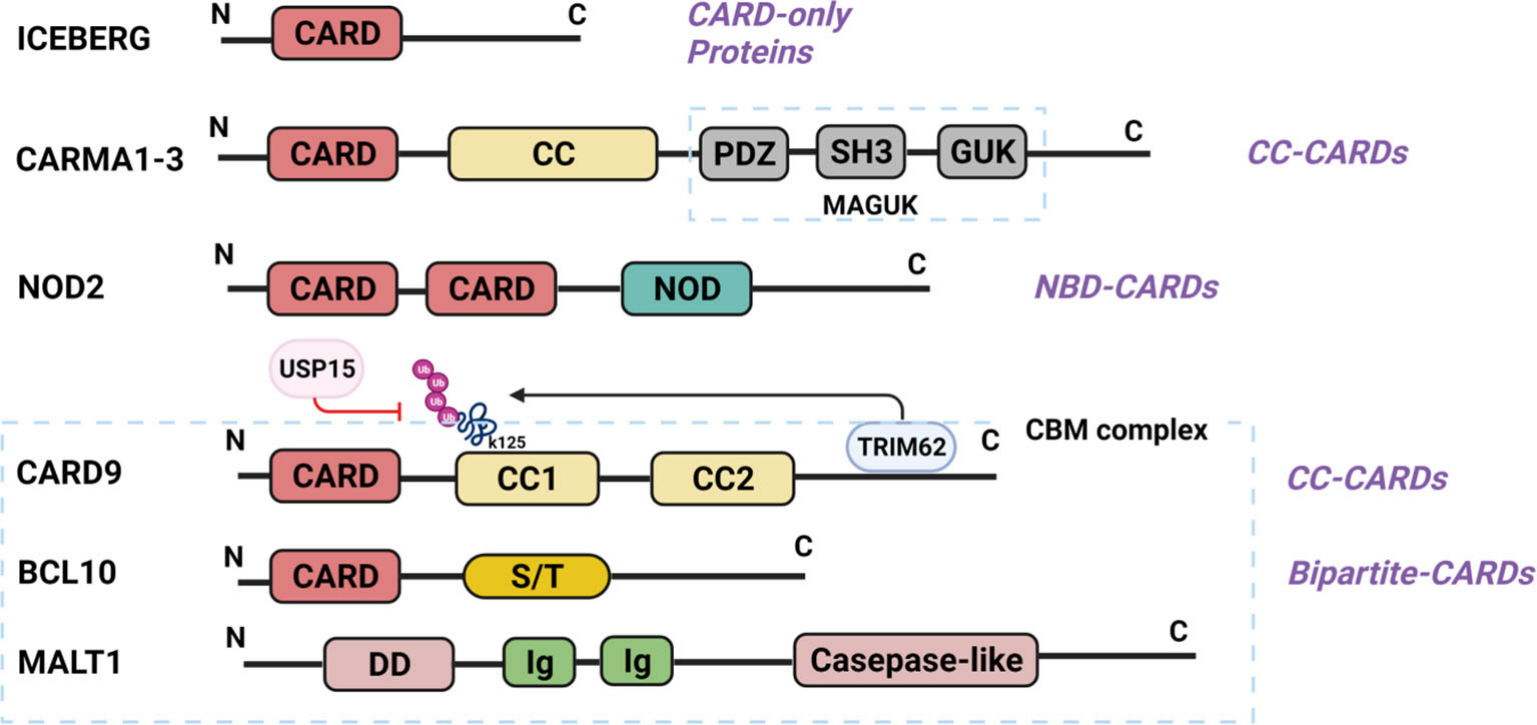
Structure of CARD-containing proteins. There are four sub-groups of CARD-containing proteins, and representative protein structure for each sub-group is shown. ICEBERG belongs to CARD-only proteins. CARMA1-3 belongs to CC-CARDs, which consist of N-terminal CARD, C-terminal CC domains and MAGUK domain (PDZ, SH3 and GUK domain). NOD2, one of the NBD-CARDs, is characterized by two CARD domains at N-terminal and a NOD domain at C-terminal, and can bind with CARD9 *via* the CARD domain. CARD9 contains a CARD domain at N-terminal and two CC domains at C-terminal, thus belonging to the CC-CARDs. Ubiquitination at K125 is through TRIM62-mediated binding with CARD9 at its C-terminal, while deubiquitination of K125 is a USP15-mediated process. BCL10 comprises of a CARD domain at N-terminal and serine-/threonine (S/T)-rich region at C-terminal, belonging to the bipartite-CARDs. MALT1 consists of an N-terminal death domain (DD), immunoglobulin-like (Ig) domains, and a C-terminal caspase-like catalytic domain. CARD9 binds with BCL10 through CARD-CARD domain, and MALT1 Ig-like domains interact with BCL10 S/T-rich region, thereby forming the CPM complex. (All the figures in the review were created with BioRender.com).

CARD is a protein interaction domain of about 90 amino acids that participates in the activation or suppression of CARD-containing members of the caspase family (22). The family of CARD-containing scaffold/adapter proteins can be divided into four sub-families based on the domain structure (23) (**Figure 1**): (1) The CARD-only proteins, including ICEBERG and pseudo-ICE/COP; (2) The CC-CARDs: These proteins contain a coiled-coil (CC) domain besides a CARD domain, including CARD9, CARD10 (also called CARMA3), CARD11 (CARMA1), and CARD14 (CARMA2); (3) The NBD-CARDs: These proteins contain a nucleotide-binding domain (NBD) instead of CC motif, including CARD4 (Nod1), CARD15 (Nod2), CARD7 (NAC/DEFCAP), and CARD12 (IPAF/CLAN); and (4) The bipartite-CARDs: In addition to a CARD domain, these proteins just contain one other motif such as a protease domain, a kinase domain, a DD motif, or a PYD motif, including CARDD (RAIDD), CARD3 (RICK/RIP2/CARDIAK), BCL10 (CIPER/mE10/CARMEN/CLAP), ARC, ASC (PYCARD), CARD-8 (TUCAN/CARDINAL), and Caspase-1,-2-4,-5,-9. The proteins of CARD families function as important intracellular signaling molecules in a variety of cell functions including apoptosis, necrosis, innate and adaptive immunity, as well as inflammation (23, 24). Particularly, the CC-CARD proteins are critical to the activation of downstream nuclear factor-κB (NF-κB) and mitogen-activated protein kinase (MAPK) pathway by recruiting other CARD-containing proteins (23–26). Among the CC-CARD proteins, CARD10, CARD11, and CARD14 belong to the membrane-associated guanylate kinase (MAGUK) family. However, CARD9 lacks MAGUK structure, and only contains a N-terminal CARD and two CC domains at C-terminus.

N-terminal CARD of CARD9 can interact with the CARD domain of B-cell lymphoma/leukemia 10 (BCL10) or nucleotide-binding oligomerization domain (NOD) proteins NOD1 and NOD2 to activate NF-κB and MAPK pathway (24–26). C-terminus is an important regulatory domain for CARD9 function (**Figure 1**). Two coiled-coil domains in the C-terminus form an extensive interface with the CARD domain, which is believed to keep CARD9 in autoinhibited state prior to activation. CARD9 ubiquitination at Lys125 (K125) could interrupt the autoinhibitory interface to initiate CARD9-BCL10 assembly (27), and Zn^2+^ binding increases the stability of CARD9-CARD domain with BCL10 (28). E3 ubiquitin ligase TRIM62 is a CARD9 binding partner, which promotes ubiquitination of CARD9 at K125 to activate NF-κB signaling (9, 29). Thus, mutation of CARD9 residue K125 (K125R) or TRIM62 deficiency significantly reduces CARD9-mediated signaling (29). USP15 is a deubiquitinase that removes TRIM62-deposited ubiquitin marks at K125 (30). USP15 deficiency, therefore, could enhance CARD9-dependent signaling. Mutations in the CARD domain (from amino acids 6 to 98) or the coiled-coil domain (from amino acids 117 to 419) would impair or abolish CARD9 protein expression and function (17, 31).

CARD9 is expressed in a variety of cells with different functions as summarized in **Table 1**. For myeloid cells, including macrophages, neutrophils and DCs, the primary role of CARD9 is to regulate the production of a wide range of cytokines and chemokines, which is critically involved in local and systemic inflammation, ROS production, oxidative stress, and the development and progression of diverse diseases (15, 48). In addition, CARD9 is important for macrophage differentiation. Thus, CARD9-defective macrophages exhibit a significant decrease in the mRNA levels of inducible nitric oxide synthase (iNOS, M1 marker) and arginase 1 (Arg1, M2 markers) after stimulation with Pneumocystis (39). CARD9-mediated production of inflammatory mediators, such as tumor necrosis factor (TNF)-α, interleukin (IL)-1β and CXCL1, is critical for neutrophil recruitment (41, 42, 49). CARD9 expression in cardiomyocytes and endothelial cells has also been reported recently. CARD9 in cardiomyocytes protects the heart from I/R injury by promoting cardiomyocyte autophagy and decreasing cardiomyocyte apoptosis (6, 46). CARD9 expression was increased in endothelial cells with shear stress upon TNF-α stimulation (47). CARD9 in microglia mediates NF‐κB activation and inflammatory cytokines production after activation of triggering receptor on myeloid cell 1 (TREM-1) following I/R injury (7).

**Table 1:** The role of CARD9 in different cells.

Cell Type	Role of CARD9	Refs.
Myeloid cells	↑Productions of pro-inflammatory cytokines (TNF-α, IL-6, and IL-1β), and chemokines (CXCL1, CXCL2, and CXCL8) through NF‐κB and/or p38 activation	(32–38)
Macrophages	↑Differentiation and polarization (M1/M2)	(39)
	↑Tumor metastasis	(15, 21, 40)
Neutrophils	↑Recruitment to infected areas	(41, 42)
Dendritic cells	↑Antigen presenting, T cell responses	(35, 43–45)
Innate lymphoid cells	↑Intestinal epithelial cells proliferation	(15, 21)
Cardiomyocytes	↑Autophagy, ↓Apoptosis	(6, 46)
Endothelial cells	↑Shear stress	(47)
Microglia	↑Inflammatory cytokines production	(7)

↑, increase. ↓, decrease.

TNF-α, tumor necrosis factor alpha; IL, interleukin; CXCL, CXC-chemokine ligand.

## CARD9 Signaling Is Critical for the Production of Cytokines/Chemokines

Cytokines and chemokines are important for immune responses and inflammation. CARD9 plays an essential role in regulating the productions of a large number of cytokines and chemokines, including innate chemokines (CXCL1 and CXCL2), innate cytokines (TNF-α, IL-1β, and IL-6), and adaptive cytokines [IL-22, IL-17 and interferon (IFN)-γ] (32–38). Myeloid cells with CARD9 deficiency exhibit a decrease in NF‐κB and/or p38 activation and production of various cytokines and chemokines, consequently leading to increased microbial infections (33, 49–51).

**Figure 2:**
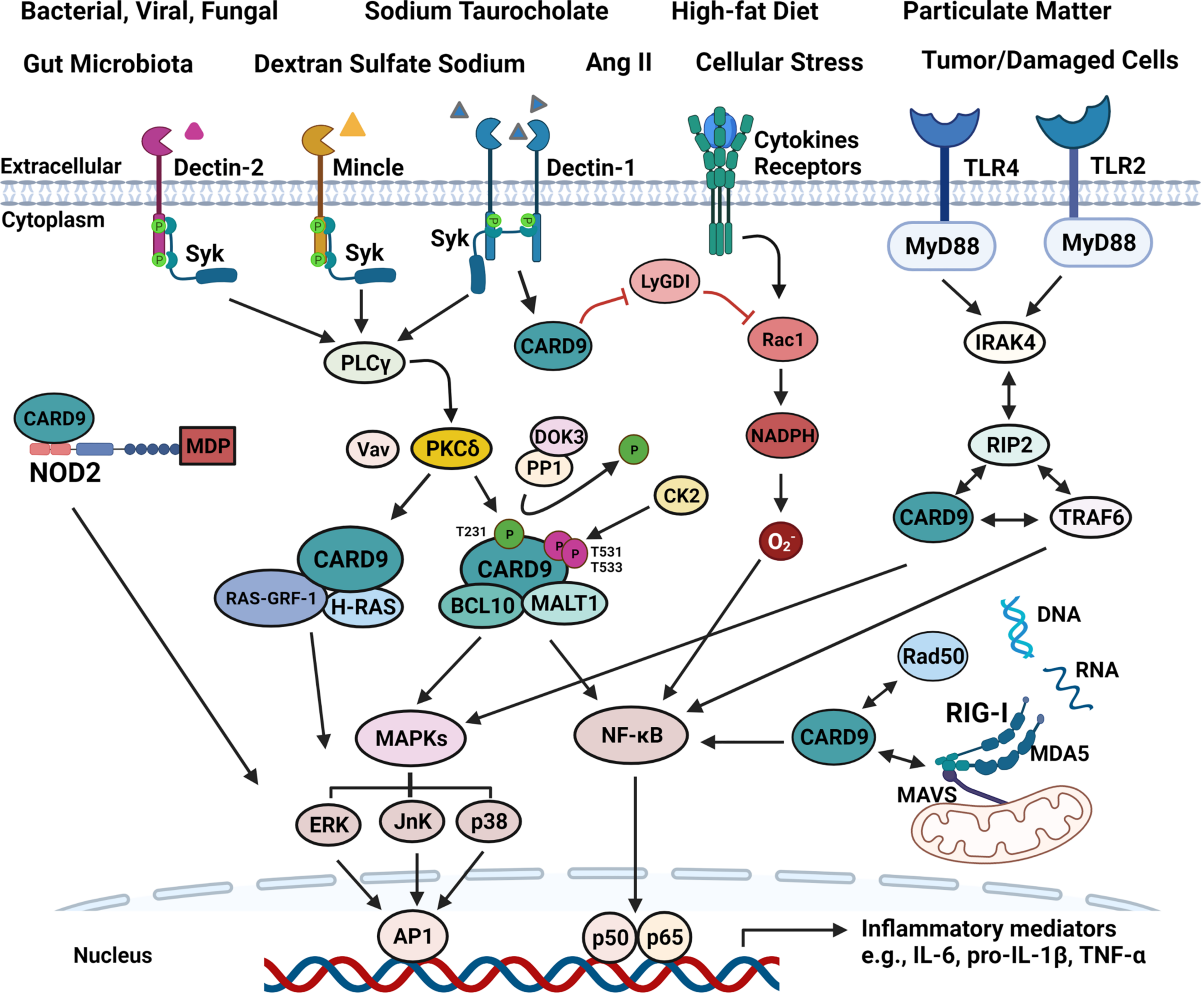
Schematic illustration of signaling pathways associated with CARD9. All four main PRRs can interact with CARD9 to promote inflammatory mediator production upon stimulation from various risk factors, such as the components of bacterial, viral and fungal, high-fat diet, Ang II, particulate matter, tumor/damaged cells. (1). CARD9 transmits extracellular pro-inflammatory signals from SyK-CLRs (Dectin-1, Dectin-2, and Mincle), and MyD88-TLRs (TLR2 and TLR4). SyK activates PLCγ, and then enhances the function of PKCδ that is critically involved in CARD9 phosphorylation at T231 with the aid of Vav proteins in the coiled-coil domain, allowing the formation of CBM complex. DOK3-PP1 complex can dephosphorylate CARD9 to keep CARD9 in an inactive state. Meanwhile, CK2 can phosphorylate CARD9 at T531/T533 to inhibit CARD9 function. The CBM complex activates NF-κB and MAPKs (ERK, JnK, p38), and subsequently increasing inflammatory cytokines production. CARD9 also binds with RAS-GRF-1 and H-RAS to activate ERK in response to Dectin-1-initiated SyK phosphorylation. RIP2 can bind with CARD9, IRAK4, and TRAF4 to activate NF-κB and MAPKs upon TLRs activation. (2). Intracellular signals, such as cytosolic MDP, DNA and RNA can be detected by NOD2, RAD50 or RIG-I-MDA5-MAVS to initiate p38, JnK or NF-κB signaling *via* CARD9, separately. In addition, CARD9 can free Rac1 from LyGDI to promote superoxide production.

As shown in **Figure 2**, C**-**type lectin receptors (CLRs) and toll-like receptors (TLRs) are transmembrane PRRs for extracellular signals. Dectin-1, Dectin-2, and Mincle are CLRs that interact with Syk to activate phosphoinositide phospholipase Cγ (PLCγ) (52). PLCγ in turn enables CARD9 to be phosphorylated at Thr231 by protein kinase Cδ (PKCδ), ultimately leading to NF-κB activation and cytokines production through CBM complex (CARD9-BCL10-MALT1) or CARD9-H-Ras-Ras-GRF1 complex (9). VAV proteins (Vav1, Vav2, and Vav3) are cytoplasmic guanine nucleotide exchange factors for Rho family GTPases, and interact with PKCδ to enhance CARD9-mediated signaling when CLRs are activated (15, 53) (**Figure 2**). Thus, Vav KO mice exhibit an increased susceptibility to fungal infections similar to CARD9 KO animals (53). Downstream of kinase 3 (DOK3) is a negative regulator for CARD9 signaling. DOK3 recruits protein phosphatase (PP)1 to keep CARD9 in dephosphorylated state, decreasing CARD9-mediated NF-κB and JnK signaling, and subsequent down-regulation of cytokines production (54, 55). Meanwhile, casein kinase 2 (CK2) can phosphorylate CARD9 at T531/T533 to inhibit CARD9 function.

Dectin-1 is the most exemplary CLR that interacts with CARD9 to activate the inflammasomes with production of pro-inflammatory cytokines and chemokines, including TNF-α, IL-1β, IL-6, IL-2, IL-10, IL-23, CXCL2, IFN-γ, and granulocyte-macrophage colony-stimulating factor (GM-CSF) upon fungal infection (8, 9). Recent studies have demonstrated that Dectin-1-induced extracellular signal-regulated protein kinase (ERK) activation can be regulated by CARD9 through linking Ras-GRF1 to H-Ras in bone marrow-derived macrophages (BMDMs) (56). Mutation of CARD9 in the CARD domain substituting the tyrosine at residue 91 with histidine (p.Y91H) impairs the CARD9-H-Ras-Ras-GRF1 complex, resulting in decreased responses of NF-κB, ERK, and GM-CSF in central nervous system candidiasis (sCNSc) (56, 57). Autoimmune regulator (AIRE) forms a transient complex with CARD9 in the Dectin-1-mediated signaling pathway that is important for the production of TNF-α (58).

Like CLRs, signaling propagation from TLRs to CARD9 involves specific intracellular signaling adaptors including MyD88 (15, 59) (**Figure 2**). When received a signal from MyD88-TLRs, the receptor-interacting-serine/threonine-protein kinase 2 (RIP2) interacts with CARD9, interleukin 1 receptor associated kinase 4 (IRAK4), and TNF receptor associated factor 6 (TRAF4), leading to the activation of NF-κB and MAPKs (15, 60). MyD88 activation and CARD9-mediated IFN-γ production are critically involved in preventing the infection of coccidioides in mice (61). Deletion of CARD9 with siRNA inhibits the signaling from TLR4 and Dectin‐1 in macrophages and reduces the phosphorylation of NF‐κB and p38 MAPKs, leading to decreased production of inflammatory cytokines (62).

Intracellularly, CARD9 also couples with NOD2 or RIG-I in cytosol for the production of pro-inflammatory cytokines and chemokines in response to cytosolic pro-inflammatory signals including DNAs or RNAs (15, 63) (**Figure 2**). NOD2 receptor, the main PRRs receptor in cytosol, interacts with CARD9 to activate p38- and JnK-signaling (51, 64). When cytosolic RNA is recognized, the association of RIG-I and melanoma differentiation-associated gene 5 (MDA5) with the mitochondrial antiviral-signaling protein (MAVS) triggers NF-κB signaling *via* CARD9-BCL10 complex (23). Rad50, a intracellular DNA-damage sensor for cytosolic DNA, couples with CARD9, leading to DNA-dependent activation of NF-κB especially in DCs (65). All in all, CARD9 plays a central role in regulating the productions of cytokines and chemokines in response to both extracellular and intracellular pro-inflammatory signals through a complex and interactive mechanisms.

## CARD9 Signaling Is Closely Associated With Adaptive Immunity

**Figure 3:**
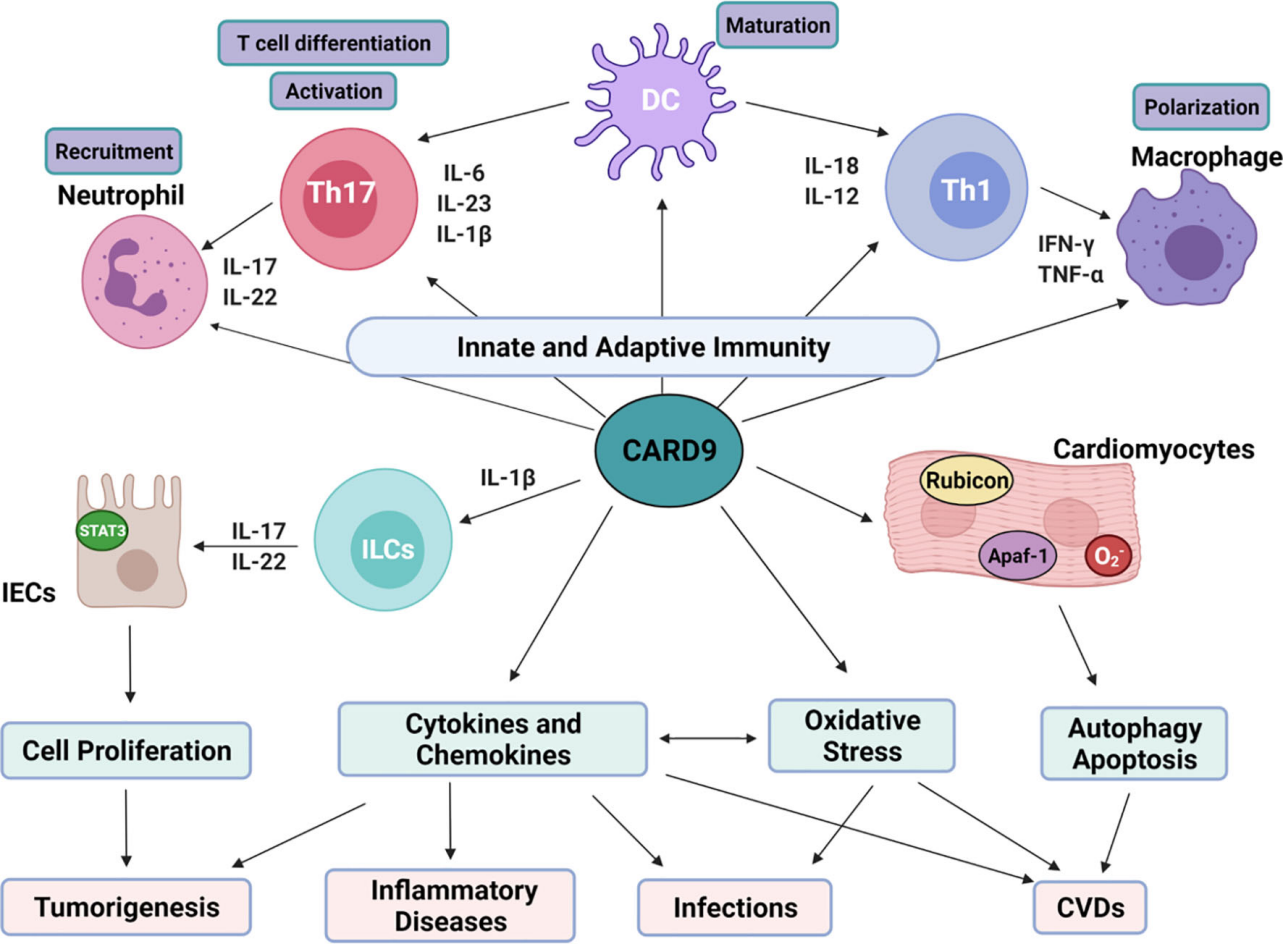
Overview on the role of CARD9 and relations with various diseases. CARD9-mediated cell proliferation, cytokines/chemokines production, oxidative stress, autophagy and apoptosis is critical for tumorgenesis, infections, inflammatory diseases and CVDs. (1). CARD9-mediated cytokines production interacts with innate and adaptive immunity. IL-6, IL-23 and IL-1β are essential for Th17 cell differentiation. IL-17 and IL-22 produced by Th17 cells trigger neutrophil recruitment. IL-12 and IL-18 from myeloid cells stimulate Th1 cell differentiation. IFN-γ released from Th1 then mobilizes and activates macrophages. The activated neutrophils and macrophages can further enhance Th1/Th17 differentiation and anti-fungal mechanisms. (2). CARD9 promotes the growth of IECs through interaction with ILCs. IL-1β from myeloid cells activates ILCs, leading to the release of IL-17 and IL-22 through CARD9-dependent signaling. After binding to its receptor on IECs, IL-22 activates STAT3, promoting IECs regeneration physiologically and tumor generation pathologically. (3). CARD9 can be activated by oxidative stress directly and indirectly. There’s crosstalk between ROS and cytokines prosunction. In cardiomyocytes, CARD9 can bind with Apaf-1 to disassociate apoptosome complex to inhibit apoptosis. CARD9 can also interact with Rubicon to promote autophagolysosome formation.

Inflammatory cytokines associated with CARD9-mediated signaling pathways are critically involved in the function of adaptive immunity as shown in **Figure 3**. IL-1β, IL-6, and IL-23 enhance the differentiation of T helper 17 (Th17) cells. IL-17 and IL-22 are the main products of Th17 cells and are important for the recruitment of neutrophils (15, 36, 66). IL-12 and IL-18 are involved in the differentiation of Th1 cells, which produce IFN-γ to mobilize macrophages (15, 36, 66). CARD9 transmits the signaling from innate immune cells to T cells, which further enhance the recruitment of neutrophils and macrophages to the areas with infections or damages. Thus, CARD9 is critically involved in various inflammatory responses. Indeed, CARD9 KO mice fail to develop adaptive Th17 cells or produce IL-17A in response to infection with Candida or Phialophora verrucosa (67–69). Recruitments of T cells, B cells, and neutrophils to the Coccidioides-infected hypodermis are reduced in CARD9 KO mice (61). CARD9(S12N) mutation facilitates Aspergillus fumigatus (Af)-induced Th2 cell-mediated RelB activation and production of IL-5 in peripheral blood mononuclear cells (PBMCs) from patients with allergic bronchopulmonary aspergillosis (ABPA) (70).

DCs, the most efficient antigen presenting cells (APCs) in immune system, link the innate immunity to the adaptive immunity. Defective CARD9 signaling in DCs impairs T cell responses, making it more susceptible to infection (15, 35, 71). Cytokines for the differentiation, activation, and expansion of Th 17 cells (IL-6, TNF-α, IL-1β, and IL-23p19) and effector cytokines from Th17 cells (IL-17 and IL-22) are significantly decreased in monocyte-derived DCs from patients with CARD9-deficiency when stimulated with Phialophora verrucosa (35). CARD9 also plays an essential role in priming hapten-specific T cells, thus mediating contact hypersensitivity (CHS) in DCs (43). It is known that haptens induce Syk activation, and promote CARD9/BCL10-dependent IL-1 secretion in DCs, thereby promoting naïve T-cell differentiation (43). Candida albicans infection in DCs induces activation of Dectin-1-Syk-CARD9 signaling to release pro-inflammatory cytokines including IL-6, TNF-α, and IL-23, which then triggers Th17 responses (71).

CARD9-mediated adaptive immune responses also play an important role in developing immunity after vaccination. Vaccine adjuvants that are used in some vaccines help to generate a stronger immune response in subjects receiving the vaccine. Vaccine adjuvants bind to PRRs to trigger robust Th17 and Th1 responses through CARD9-medated signaling pathway, a promising protective mechanism against several infections including tuberculosis (TB), chlamydial infection or malaria in preclinical studies (72, 73).

## CARD9 Signaling Enhances Oxidative Stress

ROS and oxidative stress play an important role in the regulation of cell function and disease development and progression. There are complex interactions between inflammatory cytokines and ROS production. Cytokines can trigger a significant amount of ROS formation, on the other hand, ROS can regulate the production of pro-inflammatory cytokines (13). Transmembrane NADPH oxidases (NOXs) are one of the primary endogenous enzymatic sources of H_2_O_2_, which is a major source of ROS and an important component in redox regulation. NOXs generate H_2_O_2_ in response to a variety of cytokines including IL-8, TNF-α and IFN-γ, and subsequently regulates ROS production and oxidative stress (74, 75). TNF activates NOX1 or NOX2, which converts extracellular O_2_ to ${\text{O}}_{2}^{-}$ . Subsequently, ${\text{O}}_{2}^{-}$ can be rapidly catalyzed into H_2_O_2_ by superoxide dismutases (SODs). H_2_O_2_ in turn promotes TNF production through activation of p38 and JnK signaling pathways (76, 77).

Phagocytosis and ROS production in macrophages is the initial step to microorganism elimination with critical involvement of CARD9 signaling. In macrophages, ROS formation is regulated by Rac1 through activation of NADPH complex. Rac1, a member of Rho family of small GTPases, switches between an inactive GDP-bound conformation and an active GTP-bound conformation (78, 79). GDP-dissociation inhibitor (GDI) family such as LyGDI can prevent the activation of Rac1. CARD9 interacts with the LyGDI to promote GTPase Rac1 activation during bacterial and fungal infections, allowing NADPH oxidase to produce ROS and microorganism elimination in macrophages (78). Thus, enhanced CARD9 protein expression is observed in macrophages with increased ROS production after incubation with Candida albicans (80). Zinc is considered an antioxidant and induces the synthesis of metallothionein (MT). Zinc supplement suppresses CARD9 expression with significantly reduced oxidative stress in mice on a high-fat diet (HFD) (81). However, some studies have shown that CARD9-deficient BMDMs and PMNs display normal ROS generation in response to Phialophora verrucosa infection (69, 82). ROS generation is not impaired in CARD9-deficient neutrophils isolated from a patient with invasive fungal infection and impaired neutrophil function (68). The reason(s) for the apparent inconsistency on the role of CARD9 in ROS production in macrophages and in neutrophils is unclear at this point.

## CARD9 Regulates Autophagy

Autophagy is a natural self-cleaning process through lysosomal degradation to remove damaged organelles and malformed proteins during biosynthesis or under stress (83). RUN domain Beclin-1-interacting cysteine-rich-containing (Rubicon) protein is one of the negative regulators of autophagy, which functions as a physiological feedback inhibitor of CARD9-mediated PRR signaling to avoid unbalanced pro-inflammatory responses. Following Dectin-1- or RIG-I-mediated activation, Rubicon disassembles CBM complex formation by competitively binding with CARD9, ultimately terminating PRR-induced cytokine production (84). After interacting with Rubicon, CARD9 facilitates the formation of UV-irradiation-resistance-associated gene (UVRAG)-Beclin1-phosphatidylinositol 3-kinase catalytic subunit type 3 (PI3KC3) complex and UVRAG-Vps16 complex, leading to autophagosome formation, maturation, and endocytosis (6). However, CARD9 is also reported to be able to negatively regulate autophagy. In a pressure overload mouse model by transverse aortic constriction (TAC), CARD9 KO mice exhibit a decreased protein expression of the inhibitor of κB kinase-α/β, a decreased phosphorylation of p65, and an increased expression of autophagy protein markers (LC3B II/I) in the heart (85). CARD9 deficiency prevents HFD-induced up-regulation of p38 MAPK phosphorylation, decrease of LC3BII/I ratio, and increase of p62 expression in the heart, and restores the dysfunctional myocardial autophagy (86). The reason(s) for the different roles of CARD9 signaling in autophagy is possibly due to different disease conditions. Future studies are needed to clarify the role of CARD9 signaling in autophagy.

## CARD9 and Diseases

### CARD9 Deficiency Increases the Susceptibility to Infection

CARD9 signaling is involved in innate immunity and the development of adaptive immune system, and has been recognized as an important protective mechanism against infections of selected fungi, bacteria, and viruses (14, 17). Recognition of pathogens by various PRRs initiates the signaling that is propagated *via* CARD9-mediated signaling to trigger a protective immunity against infection. CARD9 deficiency increases the susceptibility to microorganism infections both in mice and human subjects (17, 18, 87).

All CLRs, including Dectin-1/2/3 and Mincle, can interact with CARD9 against pathogenic microorganisms, especially fungi (17) (**Figure 2**). Dectin-1, Dectin-2/3, and Mincle specifically binding to β-glucans, α-mannose, and glycolipids from fungal cell walls, respectively (88). Compared to wild-type (WT) mice, CARD9 deficient mice display an impaired ability to produce local cytokines and a decrease in adaptive responses, and are more susceptible to infections with Rhizopus arrhizus, phaeohyphomycosis, Candida albicans, Cryptococcus neoformans, and Pneumocystis, resulting in elevated fungal burdens and increased mortality (32, 34, 39, 49, 89). In late phase of infection, pathogens could spread to brain, lungs, liver, spleen, kidney, and draining lymph nodes in CARD9 deficient mice (69). In addition, Dectin-1/3 and Mincle can bind with mycobacterial ligands, including trehalose-6,6-dimycolate (TDM)/trehalose-6,6-dibehenate (TDB) (90). TDM is first recognized by Dectin-3 to trigger CARD9-dependent NF-κB activation, leading to the induction of Mincle gene expression. An increase of Mincle on the cell membrane in turn promotes TDM recognition, boosting immune responses (91). Recently, using high-throughput screening of La Crosse virus (LACV) with a CLR-hFc fusion protein library, the CARD9-associated Mincle, Dectin-1, and Dectin-2 are reported to strongly interact with LACV (92). Similar to CLRs, TLRs also play a key role in host defense *via* CARD9-dependent signaling pathways, although TLRs are more specific for bacteria (59, 93) (**Figure 2**). TLR1, TLR2, TLR4, TLR5, and TLR6 are extracellular receptors that can bind with lipids or lipoproteins from microbial membrane (15, 59). Cholera toxin B subunit (CTB) exerts its pro-inflammatory effects through TLR4 and FcRγ-CARD9-mediated signaling pathway (94). TLR5 is an important signaling adaptor molecule that senses bacterial flagellin, and activates Syk and CARD9 in DCs, regulating flagellin-specific CD4^+^ T-cell responses (44).

CARD9 also couples with the NLR family in the cytosol to recognize components from bacterium, damaged cells, or dying cells (15, 95). NOD2 is considered the most representative intracellular receptor for the recognition of muramyl dipeptide (MDP) from bacteria, through binding with CARD9 to initiate the immune responses against Listeria monocytogenes, Mycobacterium tuberculosis, or other intracellular bacterial pathogens (64, 96) (**Figure 2**). CARD9 deletion also leads to a deficiency of anti-infection feedback loop against intracellular DNA virus with impaired inflammatory responses (97). CARD9 also plays an important role against RNA viruses including encephalomyocarditis virus (EMCV) and poliovirus. Cytosolic viral RNA could be recognized by the RIG-I-MDA5-MAVS axis, then triggering CARD9-mediated NF-κB activation and pro-IL-1β synthesis (98).

In addition to the well-known role in various antimicrobial mechanisms, CARD9 contributes to the pathogenesis of some diseases associated with infections. Immunity to fungal infections could lead to autoimmune diseases. Autoimmune uveitis is one of the most common causes of vision loss because of eye-specific auto-reactive T cells that disrupt immune tolerance (99). CARD9 is critically involved in Th17 polarization and Th17-associated Ag-specific responses, as well as Th1-associated responses in EAU (100). CARD9 deficient mice display a decrease in the severity of diseases and delay on the onset of EAU (99). Increased neutrophil-mediated inflammation of the airways by Moraxella catarrhalis often leads to deterioration of chronic obstructive pulmonary disease (COPD) (101). Ubiquitous surface protein A1 (UspA1) of Moraxella catarrhalis binds with PRRs such as CEACAM3 to induce Syk-CARD9 dependent activation of NF-κB pathway, leading to pro-inflammatory events including degranulation of neutrophils, ROS production, and chemokines secretion (101). The robust pro-inflammation could advance Pneumocystis or primary influenza viral pneumonia (PIVP) into acute respiratory distress syndrome (ARDS), a condition that is very difficult to treat with a high morbidity and mortality, through CARD9-dependent signaling pathway (38). During influenza virus or Pneumocystis infection, activation of macrophages and DCs induces an uncontrolled production of inflammatory cytokines/chemokines, such as IL-6, TNF and CXCL1, leading to the development of ARDS. Thus, inhibition of cytokine/chemokine production *via* targeting CARD9 signaling may provide an effective treatment option for ARDS. Indeed, influenza pneumonia mortality is dramatically decreased in CARD9 deficient mice with reduced levels of inflammatory cytokines/chemokines (38). Recently, a small-molecule BRD5529 is found to be able to bind with CARD9 and inhibit its function, attenuating p38 and pERK1 signaling and TNF-α release in macrophages stimulated with β-glucans from Pneumocystis cell wall (102).

### CARD9 Deficiency Decreases the Risk of Non-Infectious Inflammatory Diseases

Neutrophilic dermatoses with the main feature of neutrophilic infiltration are a group of noncontagious dermatological disorders, which are closely associated with respiratory comorbidities, such as asthma, COPD, and lung cancer (103). Ptpn6^spin^ mouse, characterized by persistent footpad swelling and suppurative inflammation with enhanced IL-1α-mediated signaling in neutrophils, is a well-studied mouse model for spontaneous severe inflammatory disease resembling neutrophilic dermatosis in humans (104). Enhanced IL-1α-mediated signaling in neutrophils and footpad inflammation in Ptpn6^spin^ mice were diminished in CARD9-deficient Ptpn6^spin^ mice (104). Neutrophil-specific CARD9 KO attenuates autoantibody-induced inflammation with a significant reduction of various chemokines, including CXCL1, MIP-1α (CCL3) and MIP-2 (CXCL2), as well as IL-1β (105). On the other hand, enhanced CARD9 expression is observed in severe acute pancreatitis (SAP) patients and sodium taurocholate-stimulated SAP rats compared with healthy controls, associated with increased levels of IL‐1β, IL‐6, TNF-α, and IL‐17 through CBM mediated activation of NF‐κB and p38 (106, 107). CARD9 siRNA administration reduces CARD9 expression in SAP rats, accompanied by decreases in neutrophil infiltration, NF‐κB p65 and P38 MAPK signaling, myeloperoxidase activity, and pro-inflammatory cytokines, resulting in a significant alleviation of injuries in pancreas, lung, and liver (106).

Inflammation is associated with the development of obesity and type 2 diabetes (108). CARD9 deficiency with decreased inflammation could be a protective factor in diet‐induced obesity *via* inhibition of the CARD9/MAPK pathway. In deed, CARD9 KO mice display a reduction in HFD‐induced impairment of glucose tolerance and insulin resistance with decreased expressions of p38 MAPK, JnK, and ERK compared to WT mice (109). O_2_ consumption, CO_2 _production, and heat production are also altered in CARD9 KO mice (109). CARD9 could contribute to ambient PM_2.5_-induced pulmonary injury in mice through mediating Th17 cell differentiation (110). The levels of IL-6 and IL-17A protein in bronchoalveolar lavage fluid (BALF) and their mRNA levels in lung are significantly decreased in CARD9 KO mice compared with WT mice.

### CARD9 Signaling and Inflammatory Bowel Diseases

Inflammatory bowel diseases (IBD), consisting of Crohn’s disease (CD) and ulcerative colitis (UC), are a group of gastrointestinal tract disorders that are characterized by chronic or relapsing inflammation (111). GWAS and a series of case studies suggest that CARD9 is closely associated with the pathophysiology of IBD (112–115). Some CARD9 variants could promote IBD development, while others are protective for IBD (116). The CARD9 variants associated with increased risk for IBD increase NF-κB-mediated production of cytokines (117). Conversely, the protective variant is unable to activate CARD9 since it lacks a functional C-terminal domain that is essential for the recruitment of TRIM62 to mediate CARD9 ubiquitination (117). Compounds that directly and selectively bind CARD9 to prevent TRIM62 recruitment inhibit CARD9 ubiquitination, and mimic the protective variant in IBD (117). CARD9 S12N, a missense variant where the serine is substituted by asparagine at residue 12, is a variant that is associated with increased expression of CARD9 mRNA (114, 115, 118). In contrast, CARD9 S12NΔ11 (with deletion of exon 11, Δ11) is a protective variant. This allele is reported to cause CARD9 C-terminal truncation, and provide strong protection against IBD (114, 115, 118). Thus, individuals with CARD9 S12NΔ11 variant are less likely to develop IBD. It is shown that CARD9 S12N increases TNF-α and IL-6 production in bone marrow-derived dendritic cells (BMDCs) compared to normal CARD9, while CARD9 S12NΔ11 decreases TNF-α and IL-6 production (29).

Microbiota plays a critical role in converting tryptophan into its metabolites, including indole, tryptamine, and indoleethanol, that function as aryl hydrocarbon receptor (AHR) ligands for initiation of mucosal immune responses *via* regulating IL-12 secretion (119). The microbiota from individuals with IBD displays a reduction of AHR ligands, especially in the ones with the CARD9 risk alleles linked to IBD (120). CARD9 can promote colitis recovery through up-regulation of IL-12 secretion. WT mice receiving the microbiota from CARD9 deficient mice are more susceptible to colitis due to the loss of functional microbiota (120). However, in Lyn deficiency mice, CARD9 promotes the development of colitis because of increased production of cytokines (TNF-α and IL-6) (45). Lyn kinase is a member of the Src family of tyrosine kinases (121). Deletion of Lyn leads to spontaneous development of autoimmune diseases due to hyperactivation of TLR-triggered signaling in DCs. CARD9 deletion in DCs attenuates the progress of Lyn deficiency-associated colitis in mice (45).

### CARD9 Signaling and Tumor Development and Progression

CARD9-mediated immune responses are critically involved in carcinogenesis (15, 21), as summarized in **Table 2**. The clinicopathologic analysis shows that CARD9 expression is positively correlated with tumor invasion and metastasis (15). Macrophage infiltration and excessive NF-κB activity are positively related to tumor formation and metastasis (146). It is reported that CARD9 is highly expressed in the infiltrated macrophages during carcinoma progression with enhanced production of tumor-promoting cytokines (IL-10 and IL-1α), and decreased production of anti-tumor cytokine IL-12, thus, contributing to tumor metastasis (140). CARD9 is also involved in tumor metastasis through regulation of macrophage polarization. Thus, CARD9 deficiency inhibits liver metastasis of colon carcinoma with decreased number of M2 macrophages (CD45^+^F4/80^+^CD206^+^) (140). Increased level of CARD9 mRNA is also observed in hepatocellular carcinoma *via* liver biopsies (40). There may be a sex difference in the role of CARD9 in tumor biology. It is observed that CARD9 promotes tumorigenesis of large intestines with reduced viability selectively in male mice, but not in female mice (138). In oral squamous cell carcinoma (OSCC) tissues and cells, CARD9 expression is increased with elevated levels of p-p65/p65, p-IKKα/IKKα, and p-IkBα/IkBα (139). CARD9 deletion using siRNA inhibits OSCC cell proliferation, migration, and invasion. It is known that VHL tumor suppressor protein (pVHL) is the recognition component of TRIM62, and is related to CARD9 activation in the control of NF-κB activity and tumorigenesis (141, 147). CARD9-mediated NF-κB activity is increased in renal carcinoma cells with pVHL deficiency. CARD9 deletion reduces tumorigenic potential of VHL-defective cancer cells with normalization of NF-κB activity and preserved sensitivity of cytokine-induced apoptosis (141). CARD9 signaling is important for the function of innate lymphoid cells (ILCs) after intestinal epithelial injury in mice (143). CARD9-mediated IL-1β secretion from myeloid cells is essential for the activation of ILCs and the release of IL-22 from ILCs, which in turn activates STAT3 signaling in intestinal epithelial cells (IECs) and subsequently regulate IEC proliferation (144) (**Figure 3**). With the malignant proliferation of IECs, CARD9 can promote the inflammation-associated carcinogenesis (144).

**Table 2:** The role of CARD9 in different diseases and associated mechanisms.

Diseases	Role of CARD9	Mechanisms	Refs.
Infection diseases			
Chronic mucocutaneous candidiasis, Candida meningoencephalitis, Deep Dermatophytosis, Subcutaneous or Disseminated phaeohyphomycosis, Cutaneous or invasive Aspergillosis, Exophiala disease, Corynespora cassiicola	○	↑ pro-inflammatory cytokine (TNF-α, IL-6, and IL-1β), chemokine production (CXCL1, CXCL2, and CXCL8), and Th cell responses	(17, 18, 87, 122–137)
Infection-related diseases			
Autoimmune uveitis	×	↑ Th17-associated and Th1-associated responses	(99, 100)
Neutrophilic dermatoses	×	↑ IL-1α-mediated signaling in neutrophils	(104)
COPD or ARDS	×	↑ degranulation of neutrophils, inflammatory cytokines/chemokines	(38, 101, 103)
Inflammatory bowel disease	×	S12N ↑ NF-κB–mediated cytokines production	(114, 115, 118)
	○	S12NΔ11 ↓ NF-κB–mediated cytokines production	(29, 114, 115, 118)
Colitis	○	↑ IL-12 secretion, microbiota activity	(120)
	×	↑ cytokines production in Lyn deficiency mice	(45)
Non-infectious Inflammatory Diseases			
Contact hypersensitivity	×	↑ hapten-specific T cells activation	(43)
Pancreatitis	×	↑ IL‐1β, IL-6, TNF-α and IL‐17	(106, 107)
Diet‐induced obesity	×	↑ p38 MAPK, JNK and ERK activation	(81, 109)
PM_2.5_-induced pulmonary injury	×	↑ Th17 cell differentiation, IL-6 and IL-17A production	(110)
Tumor			
Colon Tumors	×	↑ IL6 and G-CSF production, macrophage and T-cell infiltration in male mice but not in female mice	(138)
Oral squamous cell carcinoma	×	↑ p-p65/p65, p-IKKα/IKKα, and p-IkBα/IkBα	(139)
Liver metastasis of colon carcinoma	×	↑ macrophages infiltration and tumor-promoting cytokine production (IL-10 and IL-1α), ↓ anti-tumor cytokine (IL-12)	(140)
Renal carcinoma	×	↑ NF-κB activity	(141)
Lewis lung cancer	○	↓ MDSCs, ↑ CTL	(142)
Colitis-cancer	○	↓ MDSCs, ↑CTL, IL-18 and IFN-γ	(143, 144)
	×	↑ IL-1β, IL-22 and IECs proliferation	(21, 145)

○, protective. ×, predisposing. ↑, increase. ↓, decrease.

TNF-α, tumor necrosis factor alpha; IL, interleukin; CXCL, CXC-chemokine ligand; G-CSF, granulocyte-stimulating factor; IFNγ, interferon-γ; TGFβ, transforming growth factor-β; Mac-2, Galectin-3; α-SMA,α-smooth muscle actin; MCP-1, monocyte chemoattractant protein-1; NLRP3, NLR family pyrin domain containing 3. Th, T helper cells; MDSCs, myeloid-derived suppressor cells; CTL, cytotoxic T cell; IECs, intestinal epithelial cells.

However, CARD9 is also reported to prevent the development of tumors. CARD9 acts as a central regulator to ensure a long‐lasting antitumor immunity for Dectin-1-mediated activation of CD4^+^ T cells and CD8^+^ cytotoxic T-cell (CTL) (20). Myeloid-derived suppressor cells (MDSCs) can suppress CTLs to promote tumor formation (145, 148). The tumor burden is significantly increased in Lewis lung cancer mouse model after CARD9 deletion, accompanied with accumulation of MDSCs and reduction of CTL (142). In colitis-associated cancer, CARD9 increases the population of CTL by inhibiting the accumulation of MDSC and production of IL-18 and IFN-γ, and subsequently attenuates tumorigenesis (145).

### CARD9 Signaling Is Involved in Cardiovascular Diseases

It has been well established that excessive inflammation, ROS production and related oxidative stress significantly contribute to CVDs, including atherosclerosis, cardiac fibrosis, coronary artery disease, myocardial infarction, and heart failure (4, 6, 86, 149). CARD9-mediated signaling in CVDs has been well documented in several reviews (4, 16, 150). Briefly, CARD9-mediated production of cytokines and chemokines results in cardiovascular dysfunction by instigating a positive feedback loop of inflammation and oxidative stress. Therefore, inhibition of CARD9 signaling has been considered as a potential therapeutic strategy to the prevention and treatment of CVDs (4). Indeed, CARD9 deletion has been shown to prevent Coxsackievirus B3 (CVB3)-induced acute myocarditis (151), Candida albicans water-soluble extract (CAWS)-induced vasculitis (152), neointima formation of grafted veins (153), as well as TAC- or Angiotensin II (Ang II)-induced cardiac dysfunction, fibrosis, and hypertrophy (85, 154), through attenuation of NF-κB, JNK and p38 MAPKs pathway, and production of various cytokines, such as IL-6, IL-1β, TNF-α, IL-17A, TGF-β, and IFN-γ. However, deletion of hematopoietic CARD9 shows no protective effects on atherosclerosis, probably because of no significant reduction of cytokines secretion (IL-6 and TNF-α) or mRNA expressions (IL-1β, IL-10, TNF-α, and MCP-1) (149, 155).

There is a dual role of CARD9 signaling in heart I/R injury. Following I/R injury (45 minutes of ischemia followed by 24 hours reperfusion), the infarct size of CARD9 KO mice is significantly smaller than WT mice, associated with the reduction of inflammatory response and cytokines production (IL-6, TNF-α, CXCL1 and MCP-1) (156). These findings suggest that CARD9 deficiency provide a protective mechanism against heart I/R injury. However, another study shows that, when mice subjected to a shorter period of I/R injury (30 minutes of ischemia and 12 hours of reperfusion), CARD9 deficiency aggravates cardiac dysfunction in vivo by inhibiting autophagy and promoting apoptosis (6, 46). CARD9 can interact with apoptotic protease activating factor 1 (Apaf-1) *via* its CARD domain to inhibit the activation of caspase-9 and caspase-3. The reason(s) for these seemly contradictory findings is unclear, and further studies are needed to understand the inflammatory states and define the role of CARD9 signaling in each phase of cardiac I/R injury.

### CARD9 Mutations and Polymorphism Are Associated With Human Diseases

Because of the critical role of CARD9 in the production of inflammatory cytokines and chemokines during initiation of innate immunity and later adaptive immunity, mutation and genetic polymorphisms of CARD9 are reported to be correlated with a wide range of infectious diseases in humans (17–19). Autosomal recessive (AR) CARD9 deficiency has been detected in a variety of immunodeficient patients who are predisposed to invasive fungal diseases (IFD), affecting different organ systems ranging from oral mucosa, subcutaneous tissues, skin, gastrointestinal tract to central nervous system (CNS), including chronic mucocutaneous candidiasis (CMC), Candida meningoencephalitis, deep Dermatophytosis, subcutaneous or disseminated phaeohyphomycosis, cutaneous or invasive Aspergillosis, Exophiala disease, and Corynespora cassiicola infection (8, 122–137).

**Figure 4:**
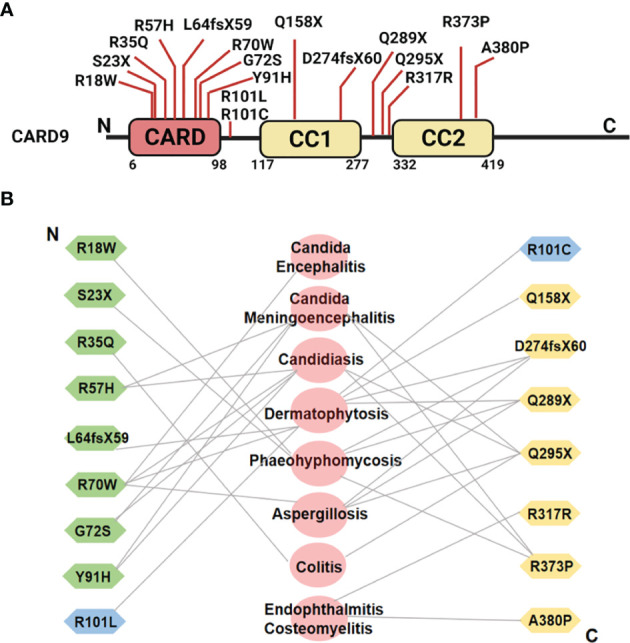
**(A)** Schematic illustration of human CARD9 protein structure and reported disease-associated CARD9 mutations. **(B)** Relation between CARD9 mutations and diseases from N-terminus to C-terminus. Symbols in green represent the CARD9 mutations in CARD domain. Symbols in yellow indicate the CARD9 mutations in coiled-coil domain. Symbols in blue indicate the CARD9 mutations in the region between CARD domain and coiled-coil domain. The diseases associated with individual mutations are shown in the middle in pink.

Human CARD9 deficiency (Q295X, a defective CARD9 protein with the glutamine at residue 295 substituting) was first reported in 2009 in a large consanguineous family with chronic mucocutaneous candidiasis (CMC) (128). Since then, more than 15 CARD9 mutations that are related to skin, mucosal, and systemic infections have been identified in different countries and populations (8, 122–137) (**Figure 4A**). These findings unambiguously highlight a critical role of CARD9 in human antifungal immunity. Importantly, one CARD9 mutation can be related with multiple diseases, and the same form of infection can be contributed by different CARD9 mutations as shown in **Figure 4B**. These mutations are associated with the absence of CARD9 protein expression or loss of function, leading to impaired productions of pro-inflammatory cytokine (TNF-α, IL-6, and IL-1β) and chemokines (CXCL1, CXCL2, and CXCL8), decreased NF-κB activation, and blunted Th1-, Th17- or Th22-associated responses to fungus-specific infection. Recently, it has been reported that hematopoietic stem cell transplantation (HSCT) from healthy sibling donors, or a combination of GM-CSF and antifungal agents successfully controlled the invasive infection, and achieved complete clinical remission in CARD9 deficiency patients with seriously fungal infection for more than one year of follow-up (157–159).

Multiple human CARD9 single nucleotide polymorphisms (SNPs) have been reported to be associated with inflammatory diseases. The association of human CARD9 rs10870077 SNP with inflammatory bowel diseases (IBD) was first described in 2008 (160). Later, the CARD9 gene haplotype CGCCA (rs4077515, rs11145769, rs59902911, rs9411205, rs4073153) is reported to be a protective factor for primary immune thrombocytopenia (ITP), Behcet’s disease (BD) and ankylosing spondylitis (AS) from Haplotype analysis and a genome-wide association study (GWAS) (161–163). It also has been suggested that the rs10870077, rs4077515, and rs10781499 variants of CARD9 be risk alleles for IBD, while rs141992399 and rs200735402 variants are protective alleles against IBD (116).

## Conclusion

CARD9 is one of the central regulators on the function of the innate and adaptive immune systems. There are extensive and complex interactions between CARD9 and other signaling molecules including NF-κB, MAPK. The primary role of CARD9 signaling in a variety of cells, particularly in myeloid cells, is to regulate inflammatory responses and oxidative stress through the expressions and productions of various important cytokines and chemokines including L-6, IL-10, IL-1β, IL-17A, TNF-α, IFN-γ, and TGF-β. CARD9-mediated signaling is critically involved in diverse cell functions and host defenses against infections of fungi, bacteria, and viruses. Abnormalities of CARD9 and CARD9 signaling are associated with a broad range of diseases especially infections, inflammatory diseases, and tumors as summarized in **Table 2**. CARD9 mutations and polymorphism are common and are associated with several human diseases especially infectious diseases and autoimmune disorders including CMC and Candida meningoencephalitis. Understanding the specific CARD9 signaling mechanisms in different cell types and relations to specific disease conditions will help defining the pathophysiology of diseases and exploring novel and effective therapy. Although significant progresses in the field of CARD9 signaling and cell function and diseases have been made for the past decade, more studies are needed to unfold the molecular mechanisms on CARD9 signaling and to define CARD9 signaling as a potential therapeutic target for disease prevention and treatment.

## Author Contributions

Conceptualization, ZL and XL. Tables and figures, XL. Writing—original draft preparation, XL. Writing—review and editing, ZL, XL, HH, and BJ. Supervision, ZL, HH, and BJ. Project administration, ZL. All authors contributed to the article and approved the submitted version.

## Funding

This work was supported by the United States NIH grants to ZL (NIH ES026200).

## Conflict of Interest

The authors declare that the research was conducted in the absence of any commercial or financial relationships that could be construed as a potential conflict of interest.

## Publisher’s Note

All claims expressed in this article are solely those of the authors and do not necessarily represent those of their affiliated organizations, or those of the publisher, the editors and the reviewers. Any product that may be evaluated in this article, or claim that may be made by its manufacturer, is not guaranteed or endorsed by the publisher.
